# History of WOFAPS (1963–2019)

**DOI:** 10.1007/s00383-020-04651-x

**Published:** 2020-04-07

**Authors:** Jose Boix-Ochoa, David Sigalet

**Affiliations:** 1 Hospital Universitario Vall d’Hebron, Barcelona, Spain; 2 Sidra Medicine, Doha, Qatar; 3 Weil Cornell, Doha, Qatar

**Keywords:** Global surgery, Low and middle-income countries, Kyoto declaration of pediatric surgery, Teaching collaboration

## Abstract

The formation of the World Federation of Associations of Pediatric Surgery (WOFAPS) was an important unifying force in the emergence of pediatric surgery as a distinct specialty. Beginning with the formation of several national societies in the early ‘60s, an early, multinational effort was created. This was in large part fostered by the International Pediatric Association (IPA), which lent logistical support from the medical pediatric community to the pediatric surgeons. In 2001, the mission of the Federation was formalized to focus on the development and education of surgeons serving children, in all parts of the world. This was articulated in the famous Kyoto Declaration of Pediatric Surgery: “Every infant and child who suffers from an illness or disease has the right to be treated in an environment devoted to their care by a pediatric medical or surgical specialist”. This vision was unique at the time and foreshadowed the major increase in advocacy activity which has emerged in recent years. While the mission has evolved with time, the present organization continues to hold true to the guiding principles of the original founders and seeks to improve the quality of “Surgical Care for the child, no matter where they live”. Education and collaboration across borders underpins the organization’s endeavors.

## Introduction

This article will present many historical details that have been drawn from lectures given by Professor Jose (Pepe) Boix-Ochoa over the years. The goal of these lectures, and this article, is to create awareness of both the challenges and successes incurred along the WOFAPS journey so that today’s pediatric surgeons can incorporate this knowledge of the “past to build the present to ensure the future” as articulated by Pepe.

Today’s WOFAPS has much to be proud of and the achievements are the result of efforts by many prominent pediatric surgeons. The journey began in early 1950 and 60s. Although the development of pediatric surgery as a distinct specialty was slow in its acceptance, the concept gained awareness when surgeons caring for children in developed countries like England, US, Canada, France, Argentina, Brazil, Australia and Japan recognized the need for a national organization as a forum for advancing the quality of pediatric surgical care. These national associations fostered development through debate and discussion of individual experiences inclusive of successes and challenges with like-minded surgeons, dealing with the unique needs of the pediatric patient. Unfortunately, in the rest of the world, the surgical care of children was still under the helm of adult surgeons, who had varying types of relationships with the corresponding pediatric medical community within that region. There was no international pediatric surgical organization, and consequently no unifying voice to develop international and universal educational and scientific guidelines for the surgical care of children at this time.

## The founding of WOFAPS

**Fig. 1:**
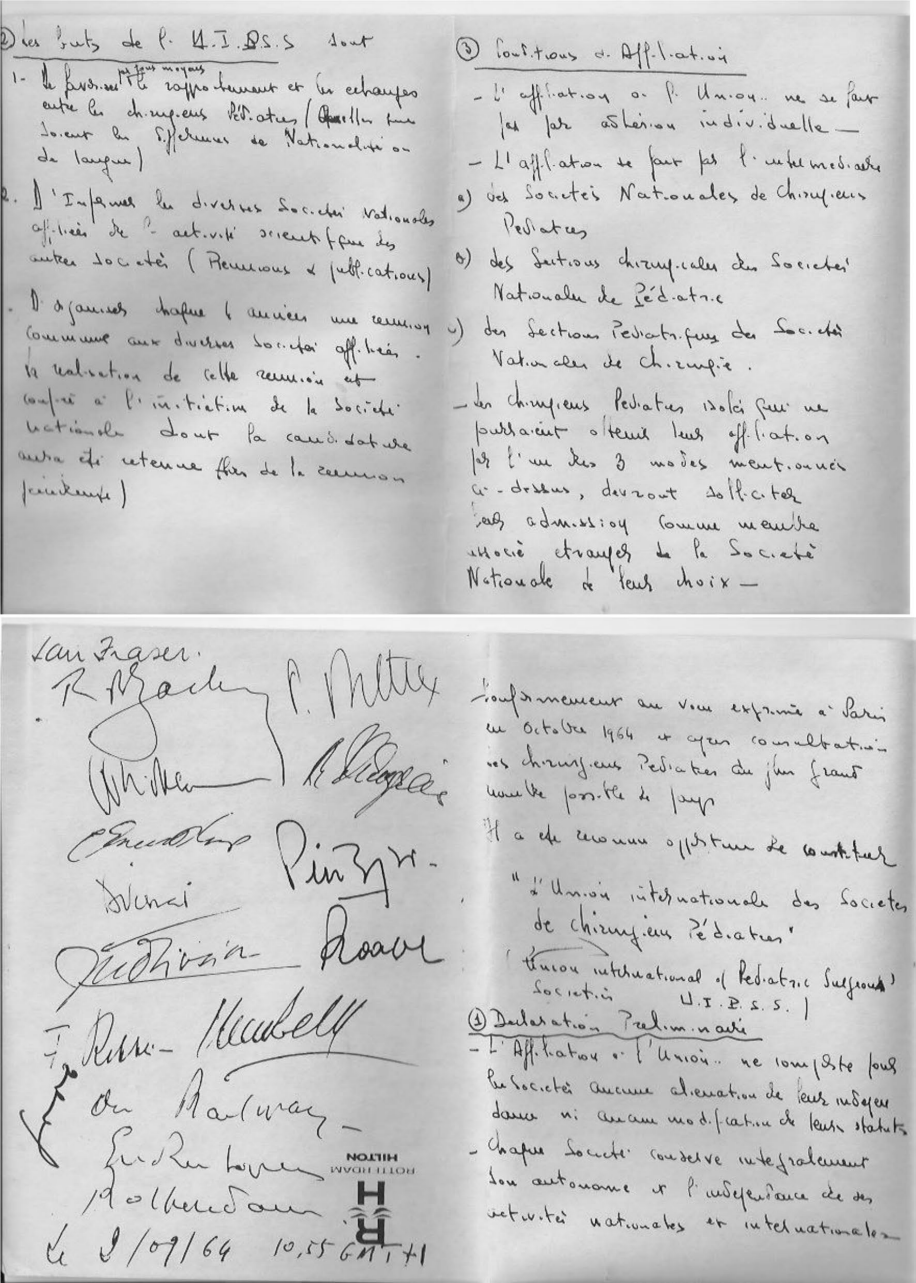
Endorsement of the proposal for the “International Union of Pediatric Surgeons” proposed by Prof Pellerin, Paris, 1963

In 1963 at the Paris Congress of the French Association, Prof. Pellerin (Hopital des Enfant, Paris) proposed the creation of the International Union of Pediatric Surgeons (IUPS) to provide support for pediatric surgeons in developing countries and to provide an international unifying voice aimed at bridging gaps in training and the level of care in. In this proposal, the IUPS articulated the aim to: (a) keep each national society fully independent, (b) improve the relationship between all pediatric surgeons, and (c) provide information on the activities of its members at a world congress every 4 years. The historic document handwritten by Prof. Pellerin is impressive; the proposal was well received and was endorsed by the signatures of 24 pediatric surgeons from different nationalities who agreed to take this proposal to their Associations (Fig. 1). Support grew and a year later in 1964 at the British Association of Paediatric Surgery (BAPS) Congress in Rotterdam, the proposal gained the support of the Francophone Societies, the Mediterranean Associations and some of the European Societies. Furthermore, it gained support in North America from Dr. Hugh Lynn (Mayo Clinic, USA) and Dr. C. Everet Koop (CHOP, Philadelphia) even though at the time the American Association of Pediatric Surgery was not yet founded.

During this time period, it is important to acknowledge BAPS as they hosted the largest pediatric surgical meeting and had a tremendous amount of influence. There was a fear by some of the leadership of BAPS that the new proposal would damage the international profile of their organization.

Despite these apprehensions, proponents of an international organization continued to work on the sidelines. In 1968, it gained support from Prof. Gral Jesus Lozaya, the founder of the Pan American Association of Pediatric surgery and Prof. Carvalho Pinto, pioneer and pillar of the Brazilian Society of Pediatric Surgeons. Loyaza and Pinto decided to link a World Symposium in Pediatric Surgery (WSPS) with the already established International Pediatric Symposium (IPA) as it would create an opportunity to present the proposal to IPA attendees. Additionally, Lozoya gained support from Professor Robert Gross (Boston Children’s, USA), who was influential in the newly formed American Pediatric Surgical Association (APSA). They recognized that the inclusion of such a large group of pediatric surgeons would create more awareness endorsing the value of an international organization. Dr. Gross shared in the enthusiasm for promoting the proposal and this was a turning point in the journey. The first World Symposium of Pediatric Surgery was held in 1968, in Mexico in conjunction with the International Pediatric Association’s symposium. This was a great success, but to build on the success of the first symposium, and to create a distinct entity for surgery, Lozaya and Pellerin made plans for the second WSPS which would follow the next IPA in 1971. The IPA was to be held in Vienna but Lozoya and Pellerin decided to host the WSPS in a separate city, Paris. This choice of venue was deliberate, recognizing that such destinations were more likely to increase attendance. Both the IPA and the WPSS were well attended demonstrating support for this type of international organization from the pediatric community.

The next step in the journey was the transition of the symposium into a proper organization representing Pediatric Surgeons from around the world. This effort was led by Jimmy Lister (Alder Hey Hospital, Liverpool), the BAPS’s general secretary (1970). Lister was a tireless organizer and a smart and flexible politician and used his influence to contact many pediatric surgeons around the world. It was implied that BAPS would lead the new organization, and further details would be discussed and developed by Lister, Beardmore (President of APSA), Professor Suruga (Japan) over the next year. The first discussion occurred in June 1972 on the sidelines at the Pan American Pediatric Surgery Congress in Tokyo. Two months later in August, this group of four met with Lozoya, Carvalho Pinto and the Presidents of South American Associations at the Pan-American Congress in Bogota. It was at this juncture that Pepe Boix-Ochoa became involved. The same year, Jimmy Lister brought a summary of these meetings to the BAPS’s Board of Governors at the Glasgow Congress and leadership of BAPS led by Nixon endorsed the proposal. Political ramifications were debated and resolved, and all associations present agreed to move forward.

The final proposal was reviewed by Jimmy Lister, Prof. Lozoya, Prof. Suruga, and Harvey Beardmore in 1973 at Lister’s home in Sheffield. They spend 3 days together after the BAPS meeting in London drafting the Constitution and By-Laws in anticipation of review by the first WOFAPS Council at the next WSPS congress (San Paulo, October 1974). It was at this meeting when the WSPS transitioned to WOFAPS.

**Fig. 2:**
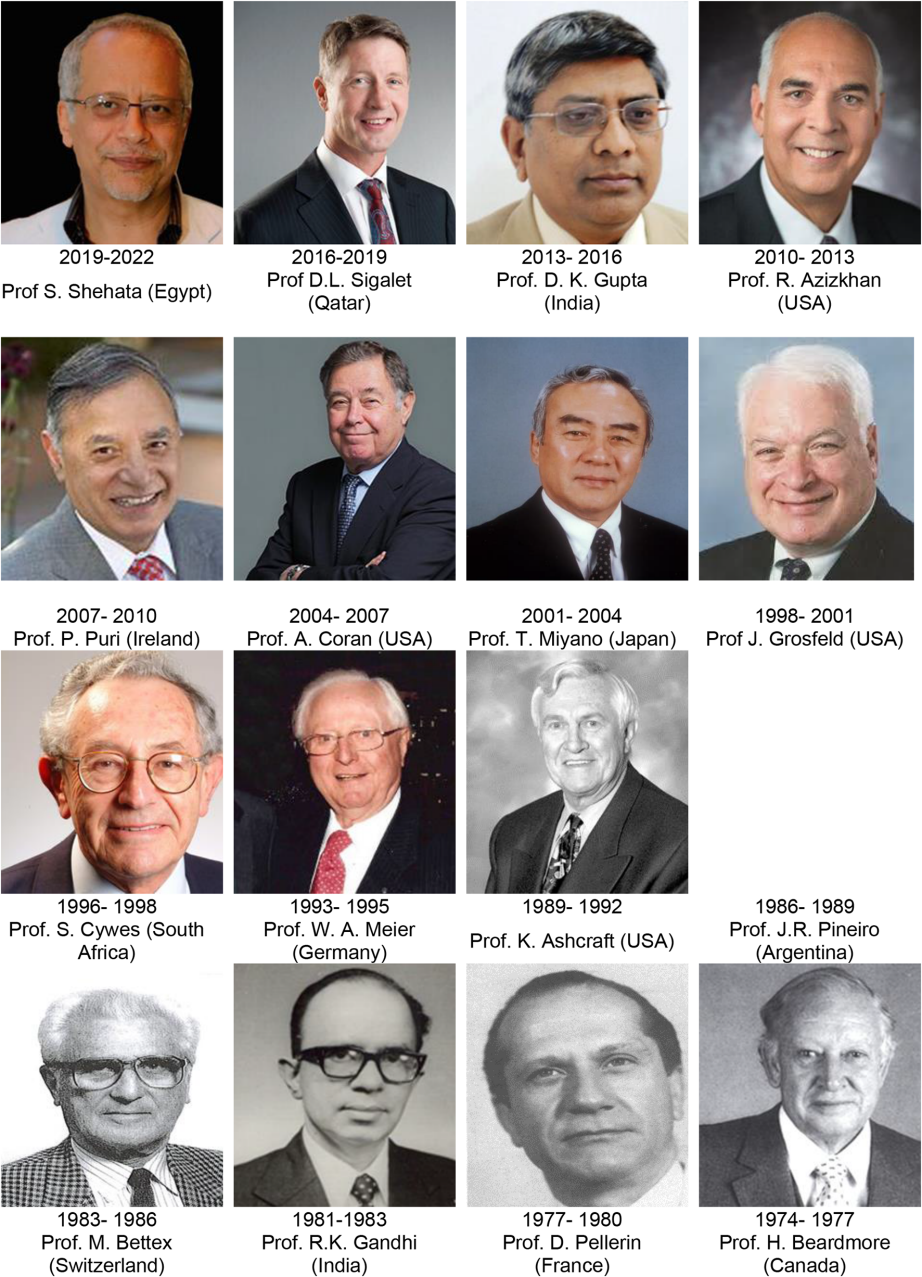
WOFAPS Presidents 1974–2022

**Fig. 3:**
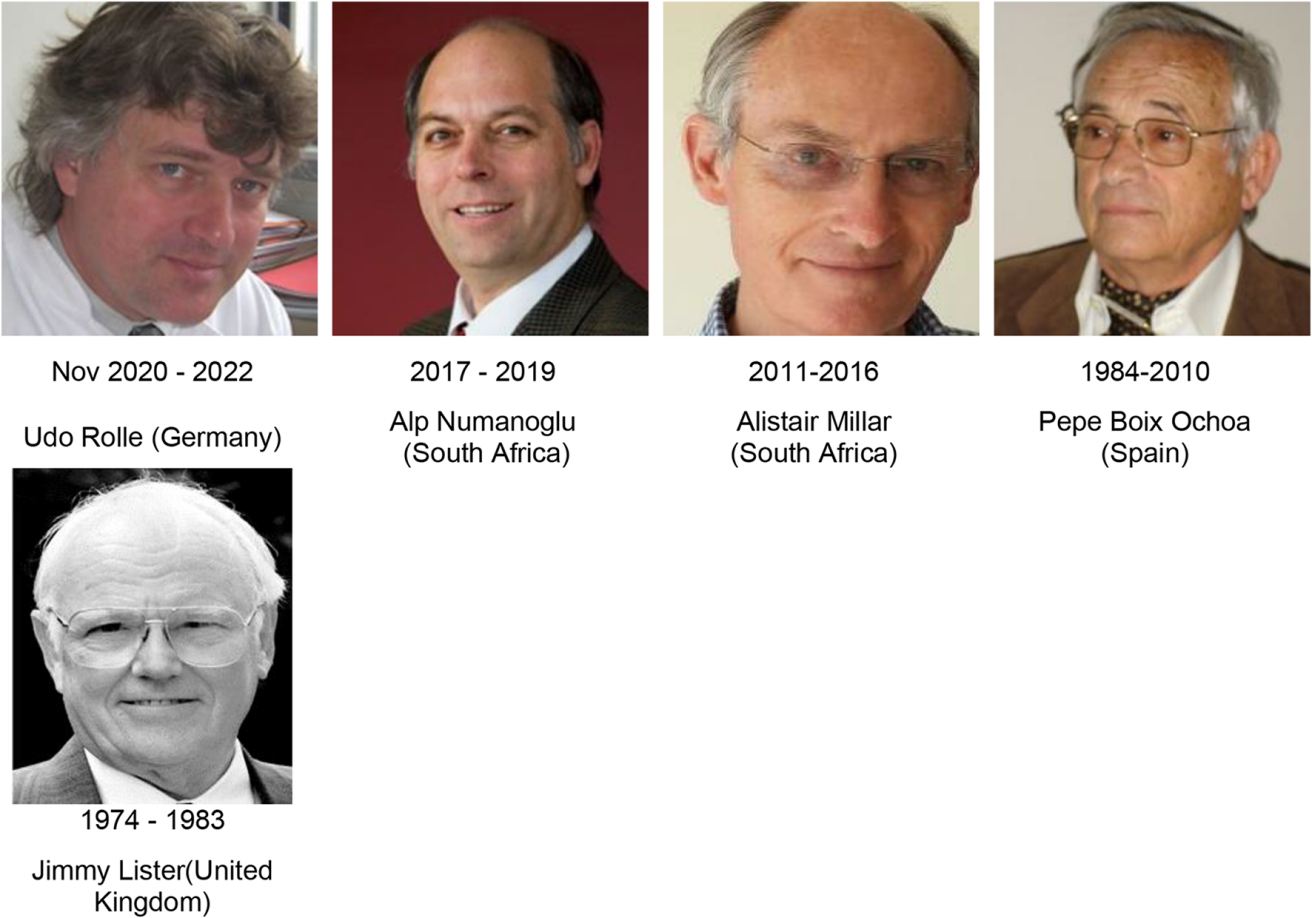
WOFAPS Secretary-Treasurers 1974–2022

After all of the efforts, debate and discussion, a smooth transition would be expected, however, the provisional agreements allocating the past President and General Secretary of BAPS to assume these posts within the new WOFAPS was not well received by the larger membership. In Sao Paulo, 27 different national associations were in attendance; the debate was resolved by offering the Presidency role to Professor Jesus Lozoya, as one of the major promoters and founders of the initial concept. He declined because of his advanced age. Ultimately, Prof. Harvey Beardmore (Montreal Children’s Hospital, Canada) who was the President of APSA was elected as WOFAPS President and Lozoya as Past-President (Fig. 2). Jimmy Lister (President of BAPS) was elected as General Secretary (Fig. 3); this was well deserved, as he was instrumental in the formation of WOFAPS. He remained as Secretary-General for 9 years (1974–1983) (Fig. 3). Unfortunately, but understandably, the BAPS president at the time, who had anticipated leading this new organization, was not happy with the outcome.

## The first WOFAPS Councils and the initial years: defining the mission

The next day, October 15, 1974, the first council meeting was held in Sao Paulo, Brazil. Fourteen of the 27 Associations represented became full members. Annual membership fees for associations were set at $100 US to entice associations to join. The Council discussed and unanimously approved a draft Constitution, the first Board of Governors and the announcement to all Societies—WHO, CIOMS, UNESCO and UNICEF—of the establishment of WOFAPS. The Constitution specified that membership to WOFAPS involved national associations and societies and not individual members. WOFAPS was established as an international body representing its various constituent associations and societies “organized exclusively for charitable, religious, educational and scientific purposes to improve the surgical care of children throughout the world”.

The goal of membership and council in the early years was to develop strong relationships with founding organizations, national pediatric surgery organizations, in addition to honing the terms of the Constitution. Under the leadership of Beardmore and Lister, the Constitution was revised and finalized for formal approval of the council at the next meeting (Barcelona, 1977).

To increase the visibility of the organization, the Executive Council decided to hold executive board meetings on the sidelines of other major congresses. The next executive WOFAPS meetings were held in Newcastle with BAPS in 1975, and in Boca Raton with APSA in 1976.

The 2nd WOFAPS World meeting was held in Barcelona in 1977, in conjunction with the 4th IPA congress of the pediatric societies. It was a great success with over 800 participants and a large delegation from Communist countries and from Africa (Table 1). Thirty-four association representatives attended the council meeting and approved the new Constitution. Representatives of the IPA and the International College of Surgeons were also included in the leadership. Prof. Denys Pellerin was elected second WOFAPS President and the general secretary and the remaining executive members were re-elected.

**Table 1:** WOFAPS'S Councils and Presidential Leadership

Meeting site	Years	President
Sao Paulo, Brazil	1974–1976	Prof. H. Beardmore (Canada)
Barcelona, Spain	1976–1980	Prof. D. Pellerin (France)
New Delhi, India	1980–1983	Prof. R.K. Gandhi (India)
Belgrade, Yugoslavia	1983–1986	Prof. M. Bettex (Switzerland)
Santiago, Chile	1986–1989	Prof. J.R. Pineiro (Argentina)
Istanbul, Turkey	1989–1992	Prof. K. Ashcraft (USA)
Hamburg, Germany	1993–1995	Prof. W. A. Meier (Germany)
Melbourne, Australia	1996–1998	Prof. S. Cywes (South Africa)
Cape Town, S. Africa	1998–2001	Prof J. Grosfeld (USA)
Kyoto, Japan	2001–2004	Prof. T. Miyano (Japan)
Zagreb, Croatia	2004–2007	Prof. A. Coran (USA)
Buenos Aires, Argentina	2007–2010	Prof. P. Puri (Ireland)
New Delhi, India	2010–2013	Prof. R. Azizkhan (USA)
Berlin, Germany	2013–2016	Prof. D. K. Gupta (India)
Washington, USA	2016–2019	Prof. D.L. Sigalet (Qatar)
Doha, Qatar	2016–2019	Prof. S. Shehata (Egypt)
Praque, Czech Republic (planned)	2019–2022	

It was during this meeting, that WOFAPS council paid tribute to IPA for their foundational role in the formation of WOFAPS but to allow for greater autonomy, elected not to join the fifth IPA in Mexico, and instead to allow the council to independently vote on the next WOFAPS venue. New Delhi was identified as the third world meeting venue with Prof. R.K. Gandhi (1980) in charge of organizing as the local host (Table 1).

The new leadership worked diligently to promote WOFAPS as the voice for pediatric surgeons and pediatric surgical patients throughout the world. New pediatric surgical societies sought membership seeing the merit and political importance of allegiance with WOFAPS.

In 1980, at the congress in Delhi, the Executive Board changed again. Prof. Ghandi was elected as president. In the fourth Council meeting held in Belgrade, Yugoslavia (1983), WOFAPS began to identify and attempt to solve important problems related to surgically ill children.

Harvey Beardmore presented his report on the contacts between developed and developing countries to ensure interchange of knowledge and helping young pediatric surgeons to train in major centers. Seventy worldwide societies (from both the developed and developing world) expressed interest but the lack of sponsors and financial support resulted in the idea remaining unfulfilled until the start of the WOFAPS Foundation some years later. The Executive Board began to develop with the formation of committees to deal with organizing the specialty.

Prof. Marcel Bettex (Berne, Switzerland) was elected President and Pepe Boix-Ochoa was again re-elected as General Secretary. (A post he would hold for 35 years!!).

In the ensuing years, under the leadership of Bettex, the organization matured, and underwent formal incorporation as a non-profit entity, in Switzerland. The following World Meeting was held in Santiago, Chile, in 1986, Jose Ricardo Piñeiro (Buenas Aires, Argentina) became the new President in 1989, with Keith Ashcraft (Kansas City, US) and J. Pinus (Sao Paulo, Brazil) as Vice Presidents. Istanbul was voted as the venue for the next Council Meeting in 1989.

## The middle years: 1989–2000 a shift to education and advocacy

Keith Ashcraft was elected president at the Istanbul council meeting of 1989. He led WOFAPS to focus on the education of young surgeons from developing countries. He tirelessly worked with the Education Committee to support and assist young pediatric surgeons in visiting major Pediatric Surgical Centers around the world as observers. He travelled to many regional centers as an ambassador for WOFAPS and hosted many young surgeons for training in Kansas City.

However, other major centers could not provide board and lodging, and candidates coming from developing countries had difficulty even arranging for transportation. The primary mechanism for overcoming these barriers was the direct support from the WOFAPS Executive, and their institutions, in hosting visiting surgeons. Dr Ashcraft also began the process of increasing communication with the membership societies, by sending out newsletters and briefings on the activities of the membership. The communication was helpful in increasing awareness, but the limiting factor continued to be the finances.

A further change began with the election of Sid Cywes in 1996 as President in Melbourne, who combined the focus on education with advocacy for regions with minimal or limited infrastructure for pediatric surgery. He actively supported and assisted WOPSEC—World Organization of Pediatric Surgeons to Emerging Countries.

Under the guidance of Prof. Cywes, the WOFAPS leadership increasing focused on assisting underdeveloped countries, particularly those in Africa, in providing medical volunteers, to advise and improve children surgical care, in addition to the counseling of traumatized patients following catastrophic events.

He also organized and assisted with training programs for post-graduates from various African countries, and was instrumental in establishing the Pan African Pediatric Surgical Association. This evolved over the years 1991–93 and resulted in the first meeting in Nairobi, Kenya with Prof. Kyamby, appointed as the first President of PAPSA (1994).

During this era, WOFAPS expanded its interaction with other major International Societies, Prof. J. Grosfeld was on the Board of Governors of the Society of Chirurgie International since 1985; at its major meeting of the International Surgical Week has always included Pediatric Surgery in all its scientific events.

WOFAPS also maintained and deepened it’s relationship with the International Pediatric Association (IPA), serving as the voice for the surgical care of children with the medical pediatric Community. Initially, WOFAPS was acknowledged as a non-voting member of the Executive Council of the IPA in 1983. Pepe Boix-Ochoa served as the WOFAPS representative for the following 20 years; through his unrelenting efforts in 2001 at the IPA meeting in Beijing, China, constitutional changes were proposed that gave WOFAPS voting stature on the Standing Committee of the Executive Council of the IPA.

The achievements of Prof. Cywe’s presidency culminated in 1998 with the South African Association of Pediatric Surgeons Congress, in Cape Town, which was combined with the Tenth WOFAPS Council. The depth of work being done by the WOFAPS Exco was reported at the Committee level, and by participating in the South African meeting, the bonds between the developed and developing countries were strengthened.

Professor Jay L. Grosfeld (Indianapolis, USA) was elected the 9th President of WOFAPS, and Takeshi Miyano (Tokyo, Japan) was elected as Vice President at the Cape Town Council meeting.

During his tenure Dr. Grosfeld was instrumental in developing the “Kyoto Declaration of Pediatric Surgery”, a milestone that outlined the guidelines for the establishment of educational requirements, and providing recommendations for the level of facilities and staffing required to care for ill and injured child and especially the newborn with congenital anomalies (Fig. 3).

**Fig. 4:**
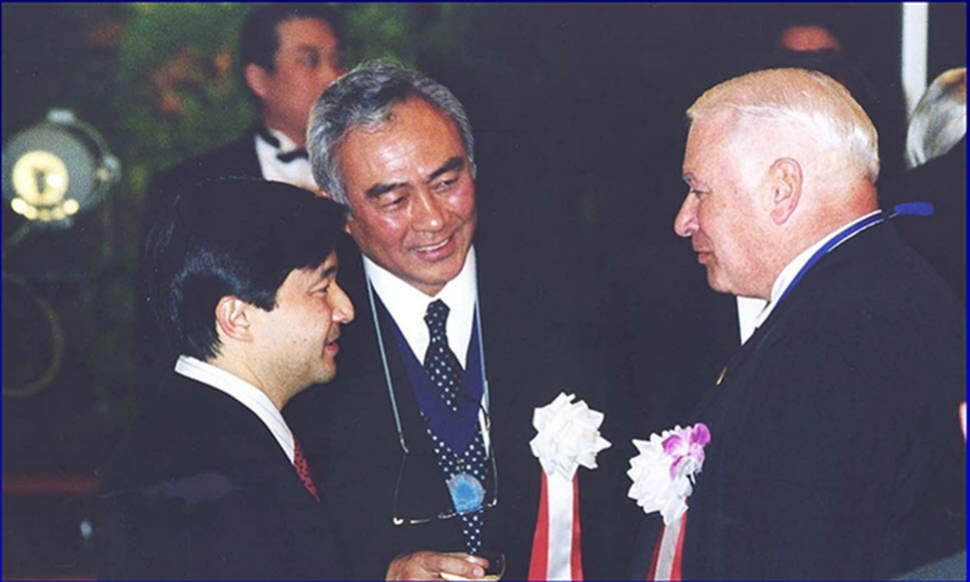
Signatories of the “Kyoto Declaration in Pediatric Surgery” Crown Prince Naruhito, Prof Takeshi Miyano and Prof Jay Grosfeld, Kyoto, 2001

The Declaration of Pediatric Surgery was voted and ratified at the 2001 meeting of WOFAPS held in Kyoto, Japan in conjunction with the Pacific Association of Pediatric Surgeons hosted by Prof. T. Miyano (Fig. 4).

The event was attended by the then Crown Prince of Japan (the current Emperor) and Professor Takeshi Miyano of Tokyo was elected the 10th President of WOFAPS.

At this time, 74 national organizations had joined the ranks of WOFAPS. A most important decision was taken by the Council in Kyoto: WOFAPS would organize the ‘World Congress of Pediatric Surgery’ to be held every 3 years, in association with the General Council meeting. The venues for the international meeting would be decided by the Association representatives in a secret ballot. The first bid for this first World Congress was won by the Croatian Pediatric Surgical Association.

The Presidency under Prof. Miyano was a memorable one; with his hardworking and tireless spirit he dedicated all his strength and worldwide connections to popularize the Declaration of Kyoto and to prepare carefully with Ivan Fattorini and his enthusiastic team for the success of the first WOFAPS Congress in Zagreb. These efforts resulted in a superb congress with an excellent scientific program and extraordinary social program. The Council elected Prof. Arnie Coran (Mott’s Children Hospital, Ann Arbor, MI, USA) as President and Buenos Aires as the next venue.

Prof. Coran’s goal was to consolidate WOFAPS and to establish a stable bureaucratic foundation for its activity. Under his guidance, the Constitution was amended, and WOFAPS was formally incorporated in Philadelphia, USA as a non-profit, tax-exempt organization, on January 18, 2007. In addition, with input from Prof. Prem Puri, and Prof. Jay Grosfeld, the WOFAPS Foundation was created, as a separate administrative entity, to carry out the charitable, educational, and scientific purposes of the Federation. Professor Grosfeld was elected the first President of the newly formed foundation. Under his guidance, a significant endowment was raised, primarily from the executive members.

## WOFAPS and the WOFAPS Foundation in the New Millenium: a path for advocacy

The World Congress of WOFAPS in 2007, organized by Prof. Juan Puigdeval from Buenos Aires held in conjunction with the umbrella Latin American organization—CIPESUR (Ciurgia Pediatrica del Cono Sur de America) and IPEG (International Pediatric Endosurgery Group) was the largest meeting ever held by WOFAPS. This meeting was a transition in programming to a more complex and more diverse educational agenda, including simultaneous sessions for the first time. There were 1500 delegates of whom 240 were residents in training and 286 delegates from the IPEG joint Meeting. Seventy-three countries were represented, with 92 member associations, representing 98% of the Pediatric Surgical Associations from around the world.

The 2007 WOFAPS Council Meeting was also notable for further refinements to the Constitution; most significantly, the composition of the Executive Committee was modified, with the addition of representatives of organizations from countries in the Middle East and Africa to assure better worldwide-representation. This composition has been maintained to the present time.

The Council voted New Delhi as next World Congress venue and Prof. Prem Puri (Dublin, Ireland) as President and Richard Azizkhan (Cincinatti, USA) as President elect.

An important practical step was the activation of the WOFAPS Foundation; Prof. Puri was able to negotiate generous funding from an Pharma Sponsor, which allowed WOFAPS to support 3–6 young pediatric surgeons each year to spend to spend three months as clinical observers in recognized and established pediatric surgical centers in various parts of the world (see Table 2). This effort and the funding has been maintained, from both the executive, and corporate donors, so that young surgeons continue to have the opportunity to travel to international centers, for the chance to work with leading surgical teams, and then to take that knowledge back to their home institutions.

**Table 2:** Kyoto declaration for the surgical care of the child

Children are not just small adults and have medical and surgical problems and needs that are often quite different from those encountered by adult physicians. Infants and children deserve the very best medical care available. Every infant and child who suffers from an illness or disease has the right to be treated in an environment devoted to their care by a pediatric medical or surgical specialist
Pediatric surgeons are specially trained physicians with extensive experience and the greatest expertise in treating infants and children of all ages (from the neonatal period through adolescence) with surgical disorders. Because of their unique training pediatric surgical specialists provide a wide range of treatment options and the highest quality care to children
Pediatric surgeons diagnose, treat, and manage children’s surgical needs including: surgical repair of birth defects, serious injuries in children (including some that require surgery), childhood solid tumors, conditions requiring endoscopy and minimally invasive procedures, and all other surgical procedures in children including ambulatory surgery
To provide the best surgical care for infants and children, complex pediatric surgical procedures should be carried out in specialized pediatric centers with intensive care facilities appropriately equipped with modern technology. In addition to the trained pediatric surgeons, these facilities should be staffed with other multidisciplinary pediatric specialists including radiologists, anesthesiologists and pathologists. These specialized centers often provide educational postgraduate training/research and should be staffed 24 h per day 7 days per week

Another important innovation of Prof Puri was the increasing the frequency of WOFAPS Executive Committee meetings with major regional meetings around the world to a twice-yearly basis, with the goal of increasing the interaction between the WOFAPS leadership and the different member organizations around the world. The first of these regional meeting took place in Hong Kong in 2009 at the time of the PAPS meeting. The second regional meeting was in Havana, Cuba in February 2010 at the time of Ibero-Americano Congress meeting. These combined regional meetings have proved to be highly successful in allowing pediatric surgeons in diverse regions to interact with WOFAPS directly. Typically the entire WOFAPS Executive participates in teaching and workshops given by the regional organization, providing an international faculty to enhance the scope of knowledge transfer.

As an extension of this goal, in 2010 a Summit meeting including the Presidents of the AAP, APSA, BAPS, CAPS, EUPSA, JSPS, PAPS and WOFAPS was held in New Delhi during the World Congress under the Chairmanship of Professor Jay Grosfeld. The purpose was to pool resources to help surgeons from developing countries to improve the surgical care of children. These efforts, with the ongoing support of the WOFAPS foundation, furthered the goal of improving surgical care of children all over the world.

Prof. Puri’s presidency ended with a very successful WOFAPS’s World Congress hosted by Prof. Gupta in New Delhi in October 2010. There were over 1000 delegates from over 80 countries, with 80 invited guest speakers, and 150 young pediatric surgeons and delegates. The General Secretary announced that more than 120 associations were registered. A delegation was invited to speak with the Prime Minister, about the development of pediatric surgery, in India, and around the world.

The Executive Council approved a number of major initiatives, including extending the selection of the venues for the World Congress out to 6 years, to allow enough time for the local committee to organize the meetings. Berlin in 2013 and Washington 2016 were elected as the next venues. Prof. Richard Azizkhan (Cincinatti USA) was confirmed as President and Prof. D. Gupta (Dehli, India) as President elect. Finally, the end of an era had arrived. Pepe Boix Ochao stepped down as General Secretary and assumed the post of honorary Secretary. Prof Alistair Millar (Cape Town, South Africa) was elected to take on this important task.

In early 2011, Prof. Azizkhan convened a WOFAPS ExCo meeting in Paris where working Committees (Scientific, Educational and Advocacy) were reorganized into a new structure with charters.

Each of these committees was led by a member of the Executive Committee (Scientific: Azzizkhan, Education: Sigalet, Advocacy: Ure) with non-EC members playing important committee roles. This provided opportunities for individuals to become engaged with WOFAPS and for the ExCo. to identify potential future WOFAPS leaders.

In particular, the Education Committee led by Prof. David Sigalet and Prof. Sameh Shehata successfully promoted numerous workshops and symposia throughout the world. They worked to encourage and promote mentor relationships between pediatric surgeons from different institutions and championed the availability of Execo members to serve as faculty for teaching and workshops in regional meetings.

During this time, two important regional meetings occurred, which followed this paradigm. The first in Tuzla, Bosnia-Herzegovina in 2011, provided a much sought after teaching experience for surgeons in the region, recovering from conflict. The second was in Cartagena de las Indias, Columbia in 2012. These proved most successful were more than 30 world known pediatric surgeons attended—paying their own costs to attend and teach, as an act of solidarity with the young pediatric surgeons from the developing countries.

Professor Azizkhan’s presidency culminated in the Congress in Berlin, October 2013, locally hosted by Prof. Jorg Fuchs. More than a thousand delegates from over 85 countries participated in this Congress. There were many highlights including eight pre-congress workshops, more than 80 invited speakers and chairpersons, four concurrent scientific sessions and the Boix-Ochoa lecture given by Prof. Alberto Peña.

Further modernization and revisions to the Constitution were done by Prof. M. Hollwarth (Graz, Austria) and his supporting committee. Prof. Devendra Gupta (Lucknow, India) was elected as President and Prof. David Sigalet (Doha, Qatar) selected as the president elect. Prof. Puri was reelected President of the WOFAPS Foundation, continuing the work of disbursing scholarships to young pediatric surgeons from LMICs. The organization pushed further into the modern era, with the appointment of Dr. Mahmoud Elfiky (Cairo, Egypt) as webmaster, and the institution of Web-based teaching and executive meetings.

The ExCo continued the pattern of holding biannual regional WOFAPS Area meetings: during the years 2013–16, Executive meetings were held with regional association conferences in Lucknow, India; Sao Paulo, Brazil; Ljubljana, Slovenia; Antalya, Turkey and Fukuoka, Japan. There was a major shift in the administrative hub of WOFAPS, to Switzerland. The financial accounts and oversight were recognized as registered in Switzerland, effective 2013. This was a major accomplishment, made possible by the hard work of Alistair Millar, the new Secretary General.

The Education Committee continued its activity, largely led by Prof. Alp Numanglu, from Cape Town. His team developed and continues to host, a series of webinars covering the major topics in pediatric surgery, which are technically excellent, serving as examples of best practise for surgeons around the world. The use of the internet as a teaching platform continues to be an important platform for providing wide scale, low-cost access to high-quality educational materials, for the WOFAPS community (www.wofaps.org).

The term ended with the 5th World Congress of Pediatric Surgery, held in Washington DC, October 5–8, 2016, with Prof Kurt Newman and Prof. Peter Kim from Children’s National as the local Organizing Chairman and the secretary respectively. About 1000 delegates from 78 countries attended the Congress.

Two important changes were initiated by the Council meeting, of the member associations. Firstly, a new category of membership was introduced; that of an individual affiliate. This allows individual surgeons to access WOFAPS educational materials, and educational support. This came about from requests for access to WOFAPS scholarships from surgeons working in countries that have no national organization. Paradoxically, the very people who would most benefit from support, from a WOFAPS fellowship, had in the past been excluded, or handicapped, by their lack of a national Society. This proposal was endorsed by the council, for the new ExCo to develop in detail.

Secondly, there was a disagreement about the representation for the European Region. In the years since the formation of EUPSA, a pattern had evolved of nominating the past president of the EUPSA as the representative for the region; this was challenged by some of the individual associations, on the grounds that this had never been formally endorsed by the members. Because of this controversy, from the floor of the meeting the proposal to have an election for these positions was made, and that the representation should be increased to two positions, to account for the large body of surgeons in that region. This was done, and in due course the representatives chosen by the member organizations were Udo Rolle, from Germany, and Zoran Bahtijarevic, from Zagreb, Croatia.

Finally, the Council elected Prof. David Sigalet (Doha, Qatar) as President, and Prof Sameh Shehata (Cairo, Egypt) as President-Elect. Alistair Millar stepped down as treasurer, and Prof. Alp Numanglu (Cape Town, South Africa) was elected as the new secretary-treasurer. Dr. Mahmoud El Fiki was re-elected as webmaster. Prague, Czech Republic was chosen as the venue for the 2022 meeting.

The new executive team, led by Prof. Sigalet, held an initial ExCo meeting in Doha 2017 in April. A small working group, led by Prof. Shehata, and the secretary, Prof. Numanglu, developed the terms of reference for the Individual affiliate membership category. Dr. El Fiki continued to improve an update the website, including the ability for fees to be paid online. This has greatly improved the financial flexibility of the organization, and there are now over 200 individual affiliate members of WOFAPS (November 2019). Dr Cohen from Melbourne arranged for a 3 year grant from Karl Storz, supporting two fellowships per year, with a preference for candidates from Oceana.

Under the leadership of Prof Sigalet, WOFAPS became a sponsoring member of two surgical consortiums, with similar goals. The Global Initiative for Children’s Surgery aims to provide a framework for developing infrastructure and human capital required to provide the appropriate surgical care for children, at the local, regional and national level. The G4 Alliance aims to develop the infrastructure for surgical care, for all ages, also at the various levels of local, district and reference center hospitals. These two efforts complement the WOFAPS mission, by helping to develop the surgical infrastructure within which pediatric surgeons can care for children, in emerging countries.

The educational group continues to host on line Webinar activity, led by Prof. Numanaglu, and the website is developing the capacity to act as source of reference material, or links to other sites. As an example, the ‘best’ of the recent webinars are available through the website at any time. This is an area of great interest to the organization; the plan is to develop this further, so that surgeons working in more isolated settings can turn to the Wofaps website as a source of trusted, evidence based reference materials. This was the topic of a meeting with the APSA leadership in Boston, April 2019, hosted by Prof. Ron Hirschl, and attended by Prof Sigalet, as well as representatives from BAPS, AAP, CIPESUR, EUPSA, and PAPS, as well as the online educational development team of APSA.

The Exco continued the pattern of holding meetings in association with regional and national associations of Pediatric Surgery; joint meetings were held in Asuncion, Paraguay (October 2017), Bucharest Romania (June 2018), Addis Abba Ethiopia (with PAPSA), Croatia (October 2018) and Xian, China (April 2019). The last meeting was notable, as this was the first WOFAPS meeting held within China.

Prof. Sigalet’s term ended with the 6th World Congress of Pediatric Surgery, held in Doha, Qatar, November 1–3, 2019, with Prof. Sigalet and Dr. Mansour Ali from Sidra Medicine the local organizing co-chairs, and Prof. Pippi Salle organized the urology component, with a pre-congress workshop given by the major leaders in pediatric urology. Over 800 delegates from 82 countries attended the Congress; notably more than 53% were from LMIC’s, which was appropriate since the theme was the optimization of care for major anomalies in an LMIC setting. Sidra Medicine (Qatar’s National Children’s Hospital) supported 100 young surgeons from LMI Countries to attend the meeting. In the Council meeting, Prof. Sameh Shehata (Cairo, Egypt) was confirmed as President, Prof. Alp Numanglu (Cape Town, South Africa) as President Elect, and Prof. Udo Rolle (Frankfurt, Germany) as Secretary-Treasurer. Antalya Turkey was chosen as the site of the 2025 meeting. Dr. Zoran Bahtijarevic was confirmed as the Chair of the foundation, and Dr. Mahmoud Elfrikey reappointed as webmaster.

At this time, WOFAPS has expanded it’s reach to include a much broader range of activity, but the goal remains the same. The words of Prof. Jay Grosfeld are as relevant today, as they were when he used this paragraph to introduce the Kyoto Declaration for the Surgical Care of the Child in 2001: "The WOFAPS objectives are to improve and maintain the standards of pediatric surgery and promote and integrate these standards throughout the world. The WOFAPS serves as a center for cooperation and interchange of information among pediatric surgical associations, societies and organizations approved by the Federation. The Federation encourages and hopes to maintain high standards of care, ethical practice, education, training, and research in pediatric surgery and its allied sciences worldwide. Over the years, the WOFAPS has matured as an organization that represents the international body of pediatric surgeons. It is recognized as a formidable group that provides support to the goals and objectives of its member organizations, educational, scientific and clinical recommendations, and an international forum that allows exchange of ideas and exposure to contemporary techniques and methodologies at its world congresses."

